# Comprehensive clinical and metabolomics profiling of COVID-19 Mexican patients across three epidemiological waves

**DOI:** 10.3389/fmolb.2025.1607583

**Published:** 2025-06-18

**Authors:** David Alejandro García-López, Joel Monárrez-Espino, Juan Carlos Borrego-Moreno, Jiamin Zheng, Rupasri Mandal, Claudia Torres-Calzada, Juan José Oropeza-Valdez, Alanne Tenório Nunes, Sergio Hugo Sánchez Rodríguez, Jesús Adrián López, Blanca Estela Calzada Rodríguez, David S. Wishart, Yamilé López-Hernández

**Affiliations:** ^1^ Metabolomics and Proteomics Laboratory, Academic Unit for Biological Sciences, Autonomous University of Zacatecas, Zacatecas, Mexico; ^2^ Departamento de investigación en salud, Hospital Christus Muguerza Chihuahua, Chihuahua, Mexico; ^3^ Departmento de Epidemiología, Hospital General de Zona #1, Instituto Mexicano del Seguro Social, Zacatecas, Mexico; ^4^ Department of Biological Sciences, University of Alberta, Edmonton, AB, Canada; ^5^ Centro de Ciencias de la Complejidad, Human Systems Biology Laboratory, Universidad Nacional Autónoma de México, Ciudad de Mexico, Mexico; ^6^ Department of Veterinary Medicine, School of Animal Science and Food Engineering (FZEA), University of São Paulo, Pirassununga, Brazil; ^7^ Cell Biology and Neurobiology Laboratory, Academic Unit for Biological Sciences, Autonomous University of Zacatecas, Zacatecas, Mexico; ^8^ MicroRNAs and Cancer Laboratory, Academic Unit for Biological Sciences, Autonomous University of Zacatecas, Zacatecas, Mexico

**Keywords:** COVID-19, biomarkers, metabolomics, mass spectrometry, metabolome

## Abstract

**Introduction:**

As of mid-2024, COVID-19 has affected over 676 million people worldwide, leading to more than 6.8 million deaths. Numerous studies have documented metabolic changes occurring during both the acute phase of the disease and the recovery phase, which, in some cases, contribute to the development of long COVID syndrome.

**Aims and methods:**

In this study, we aimed to evaluate clinical, laboratory, and comprehensive metabolomic data from hospitalized COVID-19 patients during the second, third and fourth waves (Alpha, Delta, and Omicron). A targeted, fully quantitative metabolomics assay (TMIC MEGA Assay) was used to measure 529 metabolites and lipids in plasma samples. The metabolomic profiles of these patients were compared according to different and relevant factors impacting COVID-19 outcome, such as age, sex, comorbidities, and vaccination status.

**Results:**

Among the 21 classes of compounds evaluated in this study, amino acids and lipids were the most dysregulated when comparing age, sex, comorbidities, vaccination status, and the different epidemiological waves. This is the most comprehensive analysis in Mexico providing absolute quantitative data for 529 metabolites and lipids measured in hospitalized COVID-19 patients, which could be used to monitor their metabolic status and clinical outcomes associated with COVID-19 infection or with long COVID syndrome.

## 1 Introduction

COVID-19 (Coronavirus Disease 2019) is an infectious disease caused by the SARS-CoV-2 virus (Severe acute respiratory syndrome coronavirus 2) (Khan et al., 2020). The disease rapidly spread worldwide and was declared a pandemic by the World Health Organization (WHO) in January 2020 (Loza et al., 2023). As of mid-2024, SARS-CoV-2 has infected more than 676.6 million people and caused more than 6.8 million deaths globally (Dong et al., 2020). In Latin America, more than 193 million cases and 2.9 million deaths have been reported, with Mexico accounting for over 7.6 million infections and approximately 334,000 deaths. This made the fourth most affected country in Latin America and the nineteenth worldwide (Organization PAH, 2023).

Systematic reviews and meta-analyses of metabolomic studies on COVID-19 have identified several consistent biomarkers associated with the disease’s progression, severity, and outcomes. These metabolite biomarkers provide insights into the metabolic disruptions caused by SARS-CoV-2 infection, reflecting various biological processes affected by COVID-19, such as immune response, inflammation, energy metabolism, oxidative stress, and liver dysfunction. Their consistency across different studies suggests their potential use in understanding disease mechanisms, predicting disease severity, and developing therapeutic strategies. The most frequently reported metabolomic biomarkers across various systematic reviews include amino acids and polyamines (tryptophan, kynurenine, glutamine, glutamate, arginine, ornithine, phenylalanine, tyrosine, branched-chain amino acids [BCAAs], and spermine, spermidine, among others); phosphoethanolamines (PE.O 18:0/18:1 and PE. P 16:0/18:1); lipids (lysophosphocholines [LPCs], phosphatidylcholines [PCs], free fatty acids [FFAs], and sphingolipids, such as some hexosylceramides); energy metabolism markers, including sugars and derivatives (glucose, arabinose, maltose, ribose, lactate); oxidative stress biomarkers (uric acid, glutathione); and bile acids (primary and secondary bile acids) (Pang et al., 2021; López-Hernández et al., 2021; Bourgin et al., 2023; Danlos et al., 2021; Lodge et al., 2023; Bruzzone et al., 2023).

Several epidemiological waves of COVID-19 have been reported worldwide. The exact number can vary depending on the country or region, but most of them experienced at least four to six major waves, driven by the emergence of new variants and changes in public health measures. In Mexico, the second wave (late 2020 to early 2021) was dominated for Alpha variant (B.1.1.519), and the third wave (mid-2021) was dominated by the Delta variant (B.1.617.2), which was more transmissible and associated with a more severe disease compared to Alpha. The Delta variant led to higher hospitalization rates, especially among unvaccinated individuals, and showed some ability to partially evade immunity from previous infection or vaccination, contributing to higher transmission rates globally. The fourth wave (late 2021 to early 2022) was driven by the Omicron variant (B.1.1.529) and its subvariants. Omicron was highly transmissible—significantly more than Delta—but generally associated with milder disease, especially in vaccinated individuals. Omicron exhibited a much greater ability to evade immunity from both past infection and vaccination, leading to high numbers of breakthrough infections and reinfections. This variant caused a large spike in cases worldwide, but with relatively lower rates of severe disease, hospitalizations, and deaths in vaccinated populations (Loza et al., 2023; Yang et al., 2024). However, most research characterizing the plasma metabolome of COVID-19 patients aimed at finding predictive biomarkers was conducted with the second circulating SARS-CoV-2 variant (Alpha).

The combined impact of more targeted therapies, increased vaccination rates, changes in hospital procedures, natural immunity from prior infections, and the persistence of significant metabolic alterations (leading to more comorbidities) even 2 years after the initial infection are all factors that collectively influence the plasma metabolome of patients across different waves of infection.

In the present manuscript, we aimed to evaluate a significant number of clinical characteristics, routine laboratory tests, and metabolomic features (totaling ∼580 measurements) collected from 42 patients hospitalized during the third and fourth COVID-19 waves in Mexico. We provide a broad description of the general clinical and metabolic state of these patients during active infection and treatment. Targeted metabolomics was performed using the TMIC MEGA Assay, providing quantitative values for 529 metabolites and lipids (Zhang et al., 2024). Patients from the third and fourth waves were compared, as well as those from the second COVID-19 wave recruited in the same hospital during 2020. Additionally, we compared key factors influencing COVID-19 outcomes, such as comorbidities, age, sex, and vaccination status. To our knowledge, this is the first study conducted in Mexico with such a comprehensive metabolomics approach to characterize moderate to severe COVID-19 in different epidemiological waves.

## 2 Materials and methods

### 2.1 Patients’ enrollment and sample collection

Ninety-three patients exhibiting clinical symptoms of COVID-19 were admitted to the Respiratory Triage Unit at the Hospital General de Zacatecas No. 1 of the Instituto Mexicano del Seguro Social (IMSS) between October 1, 2021 and February 23, 2022. Among these, 42 patients tested positive for SARS-CoV-2 via RT-qPCR tests. These patients were hospitalized for a maximum of 21 days and included in the present study. Blood samples for plasma analyses were collected within 2 days (on average) of admission by brachial venipuncture using BD Vacutainer® Tubes with EDTA followed by centrifugation at 2500 x g during 15 min at 4°C. We processed the samples no longer than 30 min after extraction, following recommendations from the ISO 23118:2021 (Thachil et al., 2024). For the study, we arbitrarily grouped the patients according to 1) vaccination status (vaccinated and non-vaccinated); 2) survival (survivors and non-survivors); 3) respiratory function impairment (pneumonia and non-pneumonia); 4) sex (male and female); 5) age (< 60 years and >60 years); and comorbidities (obesity/diabetes and non-obesity/diabetes), since these have been the variables that have systematically shown correlation with COVID-19 outcomes in most of the countries. The clinical information was retrieved under institutional authorization from electronic medical records using the IMSS databases and was stored in a password-protected database (Table 1). Additionally, we compared three of the epidemiological waves described in Mexico, classifying the patients according the criteria defined by Loza et al. (2023) for each circulating viral variable: Alpha (B.1.1.519): late 2020 to early 2021; Delta (B.1.617.2): mid-2021; Omicron (B.1.1.529): late 2021- early 2022. Metabolomics data from 82 patients measured in a previous study (analyzed with the TMIC PRIME assay) (López-Hernández et al., 2021) were used as reference for the epidemiological wave Alpha. This study was conducted in accordance with the Declaration of Helsinki (World Medical, 2001), with the experimental protocols approved by the IMSS research and ethics committees (registration number R-2022–3301-038 and R-2020-785-068). All participants were informed in writing about the collection of their samples for research purposes and given the right to refuse participation.

**TABLE 1:** Baseline clinical characteristics of the study population, for each stratification.

Variable	Vaccinated n = 25/non-vaccinated n = 17	Survivors n = 23/non-survivors n = 19	Pneumonia n = 27/no pneumonia n = 15	Male n = 22/female n = 20	<60 years n = 24/> 60 years n = 18	O + D n = 10/non-O + D n = 9
Age, x̄ ±SD (years)	56 (47.5–71)/53 (45–74.5) p = 0.65	60.4 ± 16.1/55.7 ± 13.1 p = 0.31	59.2 ± 15.8/56.6 ± 13.2 p = 0.59	59.2 ± 15.7/57.3 ± 14.1 p = 0.67	**47.1 ± 7.1/73.2 ± 7.1 p < 0.0001**	**60.2 ± 13/47 ± 8.2 p = 0.0184**
Male gender, n (%)	10 (40)/12 (70) p = 0.05	10 (43)/12 (63) p = 0.20	14 (52)/8 (53) p = 0.93	**22 (100)/0 (0) p < 0.0001**	12 (50)/10 (56) p = 0.72	5 (50)/2 (22) p = 0.21
Smoking, n (%)	7 (28)/8 (47) p = 0.20	8 (35)/7 (37) p = 0.89	9 (33)/6 (40) p = 0.67	10 (45)/5 (25) p = 0.17	6 (25)/9 (50) p = 0.09	2 (20)/2 (22) p = 0.90
Sudden onset of symptoms, n (%)	4 (16)/8 (47.1) p = 0.03	6 (26)/6 (32) p = 0.69	6 (22)/6 (40) p = 0.22	9 (41)/3 (15) p = 0.06	**4 (17)/8 (44) p = 0.0486**	1 (10)/1 (11) p = 0.94
Intubated, n (%)	5 (20)/5 (29) p = 0.48	**1 (4)/9 (47) p = 0.0011**	9 (33.3)/1 (7) p = 0.052	5 (23)/5 (25) p = 0.86	8 (33)/2 (11) p = 0.09	2 (20)/3 (33) p = 0.51
Pneumonia diagnosis, n (%)	15 (60)/12 (70) p = 0.48	13 (56.)/14 (74) p = 0.25	**27 (100)/0 (0) p < 0.0001**	14 (64)/13 (65) p = 0.93	15 (62)/12 (67) p = 0.78	5 (50)/7 (78) p = 0.21
Survived, n (%)	**17 (68)/6 (35) p = 0.0366**	**23 (100)/0 (0) p < 0.0001**	13 (48)/10 (67) p = 0.25	10 (45)/13 (65) p = 0.20	11 (46)/12 (67) p = 0.18	6 (60)/5 (56) p = 0.84
Vaccinated, n (%)	**25 (100)/0 (0) p < 0.0001**	**17 (74)/8 (42) p = 0.0366**	15 (56)/10 (67) p = 0.48	10 (45)/15 (75) p = 0.051	15 (62)/10 (56) p = 0.65	10 (100)/7 (78) p = 0.11
Diabetes	15 (60)/8 (47) p = 0.41	11 (48)/12 (63) p = 0.32	**11 (41)/12 (80) p = 0.0143**	15 (68)/8 (40) p = 0.07	**10 (42)/13 (72) p = 0.0490**	**10 (100)/0 (0) p < 0.0001**
Hypertension	18 (72)/13 (76) p = 0.75	18 (78)/13 (68) p = 0.47	20 (74)/11 (73) p = 0.96	17 (77)/14 (70) p = 0.59	15 (62)/16 (89) p = 0.05	10 (100)/5 (56) p = 0.02
Obesity	13 (52)/7 (41) p = 0.49	13 (56)/7 (37) p = 0.20	14 (52)/6 (40) p = 0.46	10 (45)/10 (50) p = 0.77	12 (50)/8 (44) p = 0.77\2	**10 (100)/0 (0) p < 0.0001**
Obesity and Diabetes (O + D)	**10 (40)/0 (0) p = 0.0028**	6 (26)/4 (21) p = 0.70	5 (18)/5 (33) p = 0.28	5 (23)/5 (25) p = 0.86	6 (25)/4 (22) p = 0.83	**10 (100)/0 (0) p < 0.0001**
COPD	3 (12)/2 (12) p = 0.98	**5 (22)/0 (0) p = 0.0304**	2 (7)/3 (20) p = 0.23	3 (14)/2 (10) p = 0.72	2 (8)/3 (17) p = 0.41	3 (30)/0 (0) p = 0.07
Cardiovascular Disease	14 (56)/11 (65) p = 0.57	14 (61)/11 (58) p = 0.84	15 (56)/10 (67) p = 0.48	15 (68)/10 (50) p = 0.23	14 (58)/11 (61) p = 0.86	8 (80)/4 (44) p = 0.11
Chronic renal failure	6 (24)/8 (47.1) p = 0.12	6 (26)/8 (42) p = 0.27	**6 (22)/8 (53) p = 0.0404**	9 (41)/5 (25) p = 0.27	8 (33)/6 (33) p > 0.99	1 (10)/4 (44) p = 0.09
Fever	9 (36)/5 (29) p = 0.66	6 (26)/8 (42) p = 0.27	**12 (44)/2 (13) p = 0.0404**	8 (36)/6 (30) p = 0.66	10 (42)/4 (22) p = 0.18	4 (40)/4 (44) p = 0.84
Cough	19 (76)/9 (53) p = 0.12	13 (56)/15 (79) p = 0.12	17 (63)/11 (73) p = 0.49	14 (64)/14 (70) p = 0.99	17 (71)/11 (61) p = 0.51	7 (70)/7 (78) p > 0.99
Headache	**14 (56)/3 (18) p = 0.0129**	8 (35)/9 (47) p = 0.41	13 (48)/4 (27) p = 0.17	7 (32)/10 (50) p = 0.23	12 (50)/5 (28) p = 0.15	6 (60)/7 (78) p = 0.40
Dyspnea	18 (72)/10 (59) p = 0.37	16 (70)/12 (63) p = 0.66	**22 (81)/6 (40) p = 0.0063**	15 (68)/10 (50) p = 0.23	17 (71)/11 (61) p = 0.51	8 (80)/8 (89) p = 0.59
Diarrhea	4 (16)/3 (18) p = 0.89	3 (13)/4 (21) p = 0.49	4 (13)/3 (20) p = 0.66	3 (13.6)/4 (20) p = 0.58	2 (8)/5 (28) p = 0.09	1 (10)/0 (0) p = 0.33
Chest tightness	**10 (40)/2 (12) p = 0.0468**	9 (39)/3 (16) p = 0.09	6 (22)/6 (40) p = 0.22	**3 (14)/9 (45) p = 0.0246**	5 (21)/7 (39) p = 0.20	1 (10)/3 (33) p = 0.21
Chills	9 (36)/3 (18) p = 0.20	5 (22)/7 (37) p = 0.28	10 (37)/2 (13) p = 0.10	5 (23)/7 (35) p = 0.38	9 (37)/3 (17) p = 0.14	2 (20)/5 (56) p = 0.11
Pharyngalgia	10 (40)/4 (23) p = 0.27	9 (39)/5 (26) p = 0.38	7 (26)/7 (47) p = 0.17	6 (27)/8 (40) p = 0.38	6 (25)/8 (44) p = 0.18	2 (20)/3 (33) p = 0.51
Myalgia	15 (60)/7 (41) p = 0.23	11 (48)/11 (58) p = 0.51	17 (63)/5 (33) p = 0.06	12 (55)/10 (50) p = 0.77	**9 (37)/13 (72) p = 0.0258**	6 (60)/4 (44) p = 0.50
Arthralgias	14 (56)/6 (35) p = 0.19	11 (48)/9 (47) p = 0.98	15 (56)/5 (33) p = 0.17	11 (50)/9 (45) p = 0.74	**7 (29)/13 (72) p = 0.0057**	6 (60)/3 (33) p = 0.24
Rhinorrhea	6 (24)/1 (6) p = 0.12	3 (13)/4 (21) p = 0.49	6 (22)/1 (7) p = 0.19	3 (14)/4 (20) p = 0.58	3 (12)/4 (22) p = 0.40	4 (40)/1 (11) p = 0.15
Polypnea	1 (4)/3 (18) p = 0.14	4 (17)/0 (0) p = 0.06	4 (15)/0 (0) p = 0.12	3 (14)/1 (5) p = 0.34	2 (8)/2 (11) p = 0.76	0 (0)/1 (11) p = 0.28
Anosmya	1 (4)/0 (0) p = 0.40	1 (5)/0 (0) p = 0.36	0 (0)/1 (7) p = 0.17	1(5)/0 (0) p = 0.33	0 (0)/1 (6) p = 0.24	1 (10)/0 (0) p = 0.33
Dysgeusia	1 (4)/0 (0) p = 0.40	1 (5)/0 (0) p = 0.36	0 (0)/1 (7) p = 0.17	1 (5)/0 (0) p = 0.33	0 (0)/1 (6) p = 0.24	1 (10)/0 (0) p = 0.33
Vomit	4 (16)/2 (12) p = 0.70	4 (17)/2 (10) p = 0.53	4 (15)/2 (13) p = 0.89	1 (5)/5 (25) p = 0.058	3 (12)/3 (17) p = 0.70	0 (0)/2 (22) p = 0.11
Abdominal pain	6 (24)/5 (29) p = 0.69	7 (30)/4 (21) p = 0.49	**4 (15)/7 (47) p = 0.0245**	5 (23)/6 (30) p = 0.59	8 (33)/3 (17) p = 0.22	3 (30)/3 (33) p = 0.88
Cyanosis	3 (12)/4 (23) p = 0.32	4 (17)/3 (16) p = 0.89	**1 (4)/6 (40) p = 0.0025**	4 (18)/3 (15) p = 0.78	4 (17)/3 (17) p > 0.99	0 (0)/1 (11) p = 0.28
Discomfort	8 (32)/8 (47) p = 0.32	9 (39)/7 (37) p = 0.88	11 (41)/5 (33) p = 0.63	10 (45)/6 (30) p = 0.30	8 (33)/8 (44) p = 0.46	3 (30)/3 (33) p = 0.88
Laboratory data, x̄ ±SD or x͂ (IQR)						
Erythrocytes (1 × 10^6^/mL)	4.5 ± 1/4.2 ± 1.1 p = 0.42	4.6 ± 1/4.2 ± 1.1 p = 0.21	4.5 ± 1.1/4.2 ± 1 p = 0.33	4.6 (3.8–5.2)/4.5 (3.8–4.7) p = 0.35	4.3 ± 1.2/4.4 ± 0.8 p = 0.74	**5 ± 0.7/3.6 ± 1.2 p = 0.0096**
Hemoglobin (g/dL)	12.9 ± 3.3/12.3 ± 2.6 p = 0.53	12.9 ± 3.3/12.5 ± 2.7 p = 0.67	13 ± 3/12.1 ± 3 p = 0.37	13.3 ± 3.1/12 ± 2.7 p = 0.15	12.5 ± 3.3/13.0 ± 2.6 p = 0.58	**14.9 ± 2.1/10.3 ± 2.6 p = 0.0007**
Platelets (1 × 10^3^/mL)	248.5 ± 116.1/269.2 ± 104.3 p = 0.56	245.3 ± 91.6/270.7 ± 130.2 p = 0.47	253.4 ± 116.3/264.2 ± 102.1 p = 0.77	262.6 ± 109.4/251.3 ± 114.1 p = 0.75	258.3 ± 107/255.3 ± 118.5 p = 0.93	204 (154–244.5)/236 (208.5–341) p = 0.25
Leukocytes (×10^3^)	10.9 ± 5.3/1.4 ± 3.8 p = 0.72	10.5 ± 4.8/11.7 ± 4.6 p = 0.42	12.1 ± 5/9.2 ± 5 p = 0.06	10.4 ± 3.5/11.8 ± 5.7 p = 0.33	10.7 ± 4.3/11.6 ± 5.3 p = 0.58	12.4 ± 6/10.6 ± 5.4 p = 0.51
Lymphocytes (%)	7 (4.2–13)/8.3 (4.9–13.3) p = 0.36	7.6 (4.6–13.6)/7.7 (3.8–13.3) p = 0.78	7 (4.1–11.4)/12.1 (5.8–13.6) p = 0.22	8.2 (4.3–13.2)/7.0 (4.6–16.7) p = 0.79	**11.4 (5.7–15.5)/5.1 (4.2–7.5) p = 0.0101**	**7.3 ± 5.1/17.6 ± 10.6 p = 0.0249**
Monocytes (%)	5.6 (3.5–7.3)/4.2 (2.6–5.7) p = 0.07	5.6 ± 3.2/4.7 ± 1.7 p = 0.33	4.4 (3–6.3)/4.8 (3.5–6.2) p = 0.68	4.8 (4–6.6)/4.5(2.9–6.3) p = 0.56	5 (4.2–6.9)/4 (2.3–6.1) p = 0.11	5 ± 2/6.4 ± 3.4 p = 0.33
Neutrophils (%)	86.4 (76.5–92.1)/87 (80.8–90.2) p = 0.80	87.5 (75.5–90.9)/85.4 (80.8–93.3) p = 0.53	88.5 (78.2–92.4)/81.8 (79.3–88.5) p = 0.28	83 (79.9–90.4)/87.3 (76.3–92.5) p = 0.65	**80.8 (76.4–88.8)/89.9 (85.9–92.2) p = 0.0244**	85.1 ± 8.5/76.5 ± 13 p = 0.16
Glucose (mg/dL)	141 (101–228)/136 (118–200) p = 0.62	122 (90–224)/151 (130–210) p = 0.22	136 (97–189)/169 (119–267) p = 0.24	151 (121–215)/137 (87–211) p = 0.50	130 (93–200)/155 (126–243) p = 0.12	**213 ± 86/99 ± 30 p = 0.0017**
Creatinine (mg/dL)	0.85 (0.7–1.15)/1.5 (0.7–3.5) p = 0.17	0.8 (0.7–1.3)/1.2 (0.7–3.5) p = 0.06	1 (0.6–2)/1 (0.7–3.5) p = 0.21	**1 (0.8–3.5)/0.8 (0.6–1.2) p = 0.0262**	0.95 (0.65–2.45)/0.9 (0.7–2.45) p = 0.39	1 (0.8–1.5)/0.9 (0.6–5.4) p = 0.81
Urea (mg/dL)	**51.3 (29.5–89.2)/105 (57–173) p = 0.0219**	57.4 (30.1–99.5)/83.5 (46.7–173) p = 0.24	64.4 (30.7–121)/78.3 (49.3–173) p = 0.45	**92.5 (50.1–173)/50.5 (26.5–79.2) p = 0.0118**	74.1 (27.5–114.8)/66.4 (46.4–179.4) p = 0.29	79.2 (50.2–114.6)/50.5 (21.2–130.2) p = 0.27
Reported physiological injuries, n (%)						
Kidney injury	12 (48)/9 (53) p = 0.75	10 (43)/11 (58) p = 0.35	**8 (30)/11 (73) p = 0.0064**	12 (55)/9 (45) p = 0.54	13 (54)/8 (44) p = 0.53	4 (40)/5 (56) p = 0.50
Liver injury	**6 (24)/0 (0) p = 0.0291**	4 (17)/2 (10) p = 0.53	2 (7)/4 (27) p = 0.09	2 (9)/4 (20) p = 0.31	3 (12)/3 (17) p = 0.70	3 (30)/0 (0) p = 0.07
Vascular injury	9 (36)/11 (65) p = 0.07	9 (39)/11 (58) p = 0.22	13 (48)/5 (33) p = 0.35	10 (45)/10 (50) p = 0.77	9 (37)/11 (61) p = 0.13	2 (20)/4 (44) p = 0.25
CNS injury	2 (8)/1 (6) p = 0.79	2 (8)/1 (5) p = 0.69	1 (4)/2 (13) p = 0.24	0 (0)/3 (15) p = 0.06	1 (4)/2 (11) p = 0.39	0 (0)/1 (11) p = 0.28

Student and Mann-Whitney tests were used for continuous data and Pearson and Fisher test for nominal data. Significant values (p ≤ 0.05) are highlighted in bold.

### 2.2 Targeted plasma metabolomics analysis

A targeted, quantitative metabolomics approach was employed to analyze the samples using direct flow injection mass spectrometry (DFI-MS) combined with liquid chromatography tandem mass spectrometry (LC-MS/MS). This custom LC/DFI-MS/MS assay is able to absolutely quantify up to 721 different endogenous metabolites from 40 μL of plasma, including amino acids and amino acid derivatives, biogenic amines, ceramides, cholesterol esters, diacylglycerols, acylcarnitines, glycerophospholipids, sphingomyelins, triacylglycerols, organic acids and nucleotide/nucleosides. A more detailed list of all measured metabolites and the assay’s calibration/validation protocol is provided elsewhere (Zhang et al., 2024). To minimize for pre-analytical issues associated to sample collection and processing, this quantitative method has been consistently applied to all the samples collected in our lab since 2020 and used to analyze more than 3000 serum/plasma samples since 2020, covering a wide number of clinical metabolomics/exposomics studies worldwide. All the procedures are subjected to strict quality control parameters and NIST human plasma reference material SRM 1950 is used in each batch, with less than 20% of residual standard deviation. Therefore, biological variations are larger than the technical variations that takes place in different determinations. A full list of the metabolites analyzed can be found in Supplementary Table S1.

### 2.3 Stock solutions, internal standard (ISTD) mixtures, calibration curve standards, and quality control (QC) standards

Isotope-labeled ISTDs and chemical derivatization reagents were used for accurate metabolite quantification. Chemicals were individually weighed on a Sartorius CPA225D semimicro electronic balance (Mississauga, ON, CA) with a precision of 0.0001 g. Stock solutions with defined analyte concentrations were prepared by dissolving the weighed chemicals in appropriate solvents. Seven calibration curve standards (Cal1 to Cal7), were prepared by mixing and diluting stock solutions to covering various concentration ranges according to their known or expected normal/pathological concentrations in human samples. For amino acids, amino acid derivatives, biogenic amines, nucleotide/nucleosides and organic acids, three QC standards with different concentrations were prepared by diluting the Cal7 standard solution with the same solvents as preparing the calibration standards.

### 2.4 Sample preparation

Before analysis, samples were thawed on ice, in the dark, vortexed thoroughly for 15 s and centrifuged at 13,000 × g for 10 min. The assay required 40 μL of plasma per sample (30 μL for organic acids, 10 μL for DFI analysis and amine-containing compounds) and used a 96-well plate for high-throughput analysis. The first 14 wells were used for a double blank, three blank samples (Phosphate-buffered saline, Fisher Scientific, Ottawa, ON, CA), seven calibration solutions, and three QC samples. Two sample preparations panels with different pre-column derivatization reactions were applied: 1) phenylisothiocyanate (PITC) derivatization panel, and 2) 3-nitrophenylhydrazines (3-NPH) derivatization panel. No derivatization was required for the FIA analysis.

### 2.5 LC/DFI-MS/MS analysis

Mass spectrometric analysis was performed using an ABSciex 5500 QTrap® tandem mass spectrometry instrument (MS) (Applied Biosystems/MDS Analytical Technologies, Foster City, CA) equipped with an Agilent 1290 series UHPLC system (Agilent Technologies, Palo Alto, CA). An Agilent reversed-phase Zorbax Eclipse XDB C18 column (3.0 mm × 100 mm, 3.5 μm particle size, 80 Å pore size) with a Phenomenex (Torrance, CA) A SecurityGuard C18 guard column (4.0 mm × 3.0 mm) was used for LC-MS/MS. The analysis software was Analyst 1.7.2 (Applied Biosystems/MDS Analytical Technologies, Foster City, CA). Data analysis was performed using MultiQuantTM 3.0.3 (Applied Biosystems/MDS Analytical Technologies, Foster City, CA).

The HPLC parameters used for the LC-MS/MS analysis of the PITC panel were as follows: Solvent A was 0.2% (v/v) formic acid in water, and Solvent B was 0.2% (v/v) formic acid in acetonitrile. The gradient profile for this solvent run was t = 0 min, 0% B; t = 0.5 min, 0% B; t = 5.5 min, 95% B; t = 6.5 min, 95% B; t = 7.0 min, 0% B; and t = 9.5 min, 0% B. The column oven temperature was set at 50°C. The flow rate was 500 μL/min, and the sample injection volume was 10 μL.

For DFI-MS/MS analysis, the UHPLC autosampler was directly connected to the MS ion source using red PEEK tubing. The DFI buffer mentioned above was used as the mobile phase, with the flow rate programmed as follows: t = 0 min, 30 μL/min; t = 1.6 min, 30 μL/min; t = 2.4 min; 200 μL/min; t = 2.8 min, 200 μL/min; and t = 3.0 min, 30 μL/min. The sample injection volume was 20 μL.

For the analysis of organic acids by LC-MS/MS, the solvents used were Solvent A, 0.01% (v/v) formic acid in water and Solvent B, 0.01% (v/v) formic acid in acetonitrile. The gradient profile was as follows: t = 0 min, 25% B; t = 6.0 min, 65% B; t = 6.3 min, 90% B; t = 6.5 min, 100% B; t = 7.0 min, 100% B; t = 7.5 min, 25% B; t = 12.0 min, 25% B. The column oven temperature was set to 40°C. The flow rate was 400 μL/min, and the sample injection volume was 10 μL.

### 2.6 Statistical analysis

Central tendency (mean and median) and dispersion (standard deviation and interquartile range) measures were used for continuous data to describe and compare clinical and laboratory variables of patients between different categories; for nominal variables, frequencies and percentages were used. Shapiro-Wilk was used to assess the normality. For normally distributed data, Student t-tests were used to identify mean differences; non-parametric Mann-Whitney tests were used for non-parametric data. For nominal variables, Pearson Chi^2^ and exact Fisher tests were used to identify statistically significant differences (p ≤ 0.05). Analyses and tables were generated using GraphPad Prism version 8.0.1 for Windows (GraphPad Software, La Jolla California USA).

Metabolite analysis was performed using MetaboAnalyst 6.0 (Pang et al., 2024). Metabolites with more than 20% missing values were excluded from further analysis following the commonly recommended 80% rule (Bijlsma et al., 2006). For the remaining metabolites, values below the limit of detection (LOD) were imputed using 1/5 of the minimum positive value for each variable (Wei et al., 2018). The data were log-transformed and auto-scaled to generate appropriate Gaussian distributions. Volcano plots were used to visualize the statistical significance (p-value) of changes and the magnitude of those changes (fold change, FC). Since we were interested in capturing relatively small but meaningful changes in metabolites absolute concentrations, we defined a FC > 1.2. To adjust for multiple corrections, a False Discovery Rate (FDR) cut-off of 0.05 was defined.

Principal component analysis (PCA) and two-dimension partial least squares discriminant analysis (2-D PLS-DA) scores plots were used to compare plasma metabolite data across and between study groups; 10-fold cross validation and 1000-fold permutation tests were used to minimize the possibility that the observed separation of the PLS-DA was due to chance. Variable importance in projection (VIP) and heat maps were also plotted. Significant features were considered when having a VIP score >1.5 and FDR <0.05.

## 3 Results

### 3.1 Clinical features

Table 1 describes the main clinical characteristics of patients classified according to vaccination status, survival percentage, respiratory function impairment, sex, age, and comorbidities. The average age of the patients was 58 years, with 52.4% being male and 59.5% vaccinated prior to infection. The vaccines administered included Ad5-nCoV-S from CanSino (n = 2), BNT162b2 from Pfizer BioNTech (n = 13), Sinovac-CoronaVac from Sinovac Biotech (n = 1), ChAdOx1-S from Oxford/AstraZeneca (n = 2), ChAdOx1-S from Oxford/AstraZeneca plus BNT162b2 from Pfizer BioNTech (n = 2), mRNA-1273 from Moderna plus BNT162b2 from Pfizer BioNTech (n = 2), and three patients had a vaccination report without specifying the manufacture.

### 3.2 Targeted plasma metabolomics analysis

Table 2 describes the number of metabolites for each family evaluated, as well as the number of compounds that remained in the analysis after the filtering process. A total of 636 metabolites were quantified using the targeted metabolomics assay. After removing those metabolites with values below the limit of detection (LOD) in more than 20% of the samples, 529 metabolites remained in the analysis. For each of the studied comparisons in the third and fourth waves (vaccination status, survival percentage, respiratory function impairment, sex, age, and comorbidities), no significant clustering between each group was observed by multivariate statistics (PCA and PLS-DA, data not shown). Therefore, we focused on the univariate statistics to detect any significant variation in concentrations values that can be considered for clinical monitoring of the patients.

**TABLE 2:** Chemical classes and mtabolites included and detected by the TMIC MEGA Assay in the samples evaluated.

Chemical class	Number of metabolites included in the assay	Number of metabolites that remained in the analysis
Amino acids and derivatives	64	54
Organic acids	53	39
Biogenic amines	19	9
Nucleobases and nucleosides	22	8
Catecholamines	4	0
Kynurenine-tryptophan pathway metabolites	9	4
Ketone and keto acids	7	6
Indole derivatives	9	3
vitamins and derivatives	3	1
Sulfates	4	1
Dipeptide	1	0
Triglycerides	242	202
Phosphatidylcholines	75	75
Acylcarnitines	40	27
Cholesteryl esters	22	13
Diglycerides	44	20
Ceramides	36	12
Hexosylceramides	19	17
Lysophosphatidylcholines	14	14
Sphingomyelins	14	14
Dihexosylceramides	9	6
Trihexosylceramides	6	3
Sugars	2	2
Miscellaneous metabolites	3	3

**FIGURE 1:**
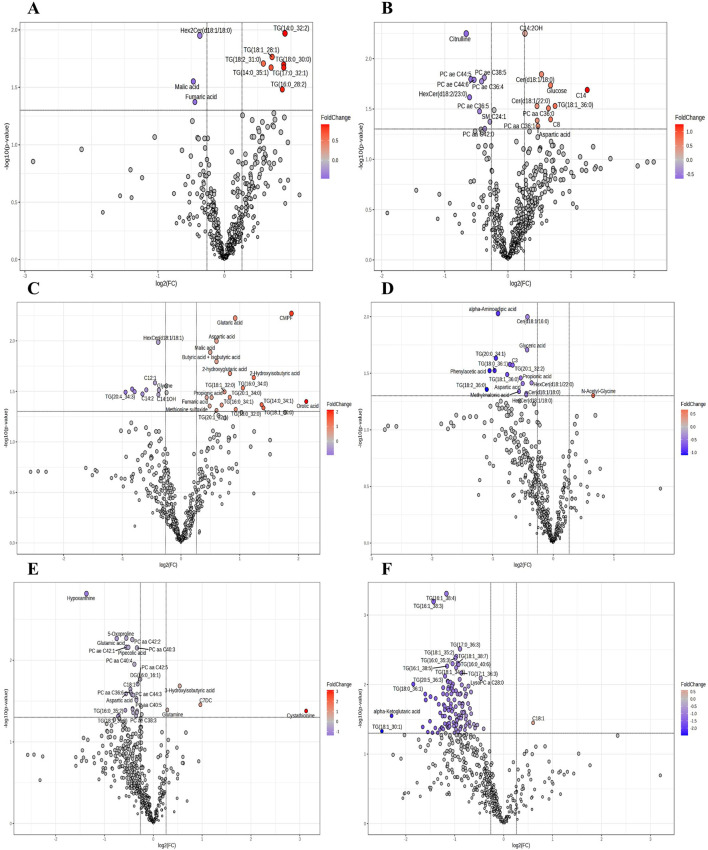
Volcano plot of the plasma metabolome among different groups of COVID-19 patients. **(A)** Vaccinated vs. non-vaccinated. **(B)** Survivors vs. non-survivors. **(C)** Diagnosis of pneumonia vs. no pneumonia. **(D)** Male vs. female. **(E)** Younger than 60 years old vs. older than 60 years. **(F)** With obesity and diabetes vs. without obesity and diabetes. Fold change (FC) threshold >1.2 and p-value ≤0.05).

Figure 1 shows the volcano plots for different group comparisons. When comparing vaccinated vs. non-vaccinated patients, 7 metabolites were upregulated, while three were downregulated (Figure 1A). The comparison of survivors vs. non-survivor showed 10 metabolites upregulated and 9 downregulated, with the following two metabolites being identified as most significantly different: acylcarnitine C14:2OH (p = 0.0056, increased, F.C. = 1.2) and citrulline (p = 0.0056, decreased, F.C. = 0.63) (Figure 1B). In patients who were diagnosed with pneumonia, 19 metabolites were upregulated while 9 were downregulated with the most significantly altered metabolites being the fatty acid 3-carboxy-4-methyl-5-propyl-2-furanpropionic acid (CMPF) (p = 0.0054, increased, F.C. = 3.68), glutaric acid (p = 0.006, increased, F.C. = 1.89) and aspartic acid (p = 0.01, increased, F.C. = 1.52) (Figure 1C).

The amino acid alpha-aminoadipic acid (p = 0.0094, decreased, F.C. = 0.53) and the ceramide Cer(d18:1/16:0) (p = 0.01, decreased, F.C. = 0.74) were the most significantly differentiated metabolites when comparing male and female patients (Figure 1D). The comparison of patients under 60 years old vs. over 60 years old showed four metabolites upregulated and 26 downregulated. The most significantly changed were the nucleobase hypoxanthine (p = 0.0015, decreased, F.C. = 0.39), 5-oxoproline (p = 0.0054, decreased, F.C. = 0.68), glutamic acid (p = 0.0054, decreased, F.C. = 0.59), along with the glycerophospholipids PC aa C42:2 (p = 0.0056, decreased, F.C. = 0.74), PC ae C42:1 (p = 0.0069, decreased, F.C. = 0.68), PC aa C40:3 (p = 0.0071, decreased, F.C. = 0.79), and pipecolic acid (p = 0.0069, decreased, F.C. = 0.70) (Figure 1E). In patients with both diabetes and obesity, only one metabolite was found to be upregulated and 119 were downregulated. 18 triglycerides were significantly downregulated (Table 3) and the glycerophospholipid LysoPC a C28:0 (p = 0.0081, decreased, F.C. = 0.72) (Figure 1F).

**TABLE 3:** The most significantly downregulated triglycerides in obesity and diabetes clustering.

Metabolite	Class	p-value	Fold change
TG (16:1_38:4)	Triglycerides	0.0005, decreased	F.C. = 0.44
TG (16:1_38:3)	Triglycerides	0.0006, decreased	F.C. = 0.37
TG (17:0_36:3)	Triglycerides	0.0031, decreased	F.C. = 0.54
TG (18:1_35:2)	Triglycerides	0.0039, decreased	F.C. = 0.51
TG (16:0_35:3)	Triglycerides	0.0042, decreased	F.C. = 0.50
TG (18:1_38:7)	Triglycerides	0.0049, decreased	F.C. = 0.52
TG (18:1_33:2)	Triglycerides	0.005, decreased	F.C. = 0.48
TG (16:0_40:6)	Triglycerides	0.0051, decreased	F.C. = 0.53
TG (16:1_38:5)	Triglycerides	0.0054, decreased	F.C. = 0.45
TG (18:1_34:3)	Triglycerides	0.0057, decreased	F.C. = 0.51
TG (17:1_36:3)	Triglycerides	0.0068, decreased	F.C. = 0.55
TG (20:5_36:3)	Triglycerides	0.0076, decreased	F.C. = 0.44
TG (18:1_35:3)	Triglycerides	0.0084, decreased	F.C. = 0.54
TG (18:0_34:3)	Triglycerides	0.0089, decreased	F.C. = 0.45
TG (16:0_37:3)	Triglycerides	0.0092, decreased	F.C. = 0.45
TG (18:2_33:0)	Triglycerides	0.0093, decreased	F.C. = 0.47
TG (18:2_32:2)	Triglycerides	0.0098, decreased	F.C. = 0.49
TG (18:0_36:1)	Triglycerides	0.0099, decreased	F.C. = 0.28

The lists of dysregulated metabolites in the different clinical or demographic clusters can be seen in Supplementary Tables S2–S7 with the metabolite classes also reported.

### 3.3 Comparison of the three different waves (Alpha, Delta, and Omicron)

**FIGURE 2:**
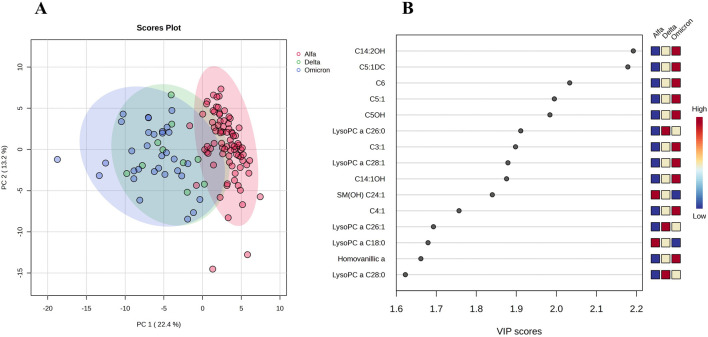
Multivariate analysis of plasma metabolome profile of patients from Alpha, Delta, and Omicron COVID-19 waves. **(A)** Principal component analysis (PCA) comparing the three epidemiological waves. **(B)** Rank of the different metabolites (the top 15) identified by the PLS-DA according to the VIP coefficient on the x-axis. The most discriminating metabolites are shown in descending order of their coefficient scores. The color boxes indicate whether metabolite concentration is increased (red) or decreased (blue).

Figure 2 shows the comparison of plasma samples from hospitalized patients during the second COVID-19 wave (Alpha) and those from the Delta and Omicron COVID-19 waves. NIST human plasma reference material, SRM 1950, as well as glucose and lactic acid values were similar in the two different batches evaluated and no *post hoc* drift correction or inter-batch normalization was required. Supplementary Figure S1 shows the PLS-DA performance validation. Seventy-five metabolites and lipids showed significant differences in this comparison. Differences were noted for acylcarnitines: C14:2OH, C5:1DC, C5:1, C4:1, C5DC, C12, C14 and C2; lysophospholipids: LysoPC a C26:0, LysoPC a C28:1, LysoPC a C26:1, LysoPC a C18:0 and LysoPC a C8:0; amino acids: arginine, methylhistidine and aspartic acid; as well as organic acids: homovanillic acid, lactic acid, citric acid, uric acid, and hippuric acid when comparing delta and omicron waves. With the exception of SMOH 24:1 and lysoPC aa C18:0, lipids were found to be increased in patients from the Delta and Omicron waves compared to those from the Alpha wave.

## 4 Discussion

This study provides the most comprehensive analysis of clinical characteristics, laboratory parameters, and targeted plasma metabolomics of COVID-19 patients in Mexico across vaccination status, age, sex, comorbid conditions, and during various epidemic waves (Alpha, Delta, and Omicron). By the absolute quantification of 529 metabolites and lipids, this study offers in-depth insights into the metabolic alterations specific to COVID-19, comparing patients based on vaccination status, comorbidities, age, sex, and epidemiological waves. Unlike previous studies that have focused on fewer metabolites or employed less precise techniques, this study provides an extensive metabolomic dataset, making it a significant contribution to understanding the metabolic disruptions associated with COVID-19 and to provide quantitative results for the clinical monitoring of patients.

The findings highlight the complex interplay between clinical features and metabolomic alterations, reflecting distinct physiological and immunological responses. While the acute phase of COVID-19 has become less dominant due to widespread vaccination, immunity (whether from vaccination or prior infection), and the emergence of milder variants, the long-term health consequences of the virus continue to be a major area of concern. Long COVID, characterized by lingering symptoms such as fatigue, brain fog, and organ dysfunction, remains a challenge for healthcare systems worldwide. Additionally, the virus’ potential to predispose individuals to other conditions such as cardiovascular diseases, diabetes, and neurological disorders underscores its ongoing relevance. Moreover, the virus may still cause localized outbreaks or seasonal surges, and new variants could continue to emerge, making COVID-19 a continuing public health issue. Therefore, the quantitative measurement of metabolites affected during the acute and recovery phases of the disease provides valuable insights for monitoring health outcomes and mitigating disease impact.

There are several metabolomic studies on COVID-19 that have consistently identified several disrupted metabolic pathways and metabolites linked to the disease’s progression and severity in line with our findings. Key metabolic pathways affected include amino acid metabolism (such as arginine, proline, tryptophan, and glutamine metabolism), energy metabolism (including the TCA cycle and glycolysis), lipid metabolism (sphingolipids, phosphatidylcholines), and bile acid metabolism. Amino acids like tryptophan, kynurenine, and glutamine, along with fatty acids such as acylcarnitine, and sphingolipids, have been consistently reported as significantly altered in COVID-19 patients. These alterations are closely associated with inflammation, immune dysregulation, and mitochondrial dysfunction. In particular, the tryptophan/kynurenine pathway has been repeatedly implicated, with elevated kynurenine and reduced tryptophan levels associated with the severity of the disease. Additionally, acylcarnitines, and amino acid markers have been linked to mitochondrial dysfunction and cellular energy imbalance (Elgedawy et al., 2024; Bi et al., 2025; Costanzo et al., 2022; Buyukozkan et al., 2022; Lee et al., 2024; Mallol et al., 2025). For instance, increased acylcarnitine levels, particularly C14:2OH, are associated with poor outcomes, while metabolites such as citrulline and aspartic acid are linked to better survival. This provides a basis for using targeted metabolomic profiling to monitor COVID-19 progression and develop early intervention strategies.

The metabolomics method employed here detects and quantifies up to 721 metabolites covering more than 20 different chemical classes. The assay has been validated and used to analyze more than 3000 serum/plasma samples since 2023, covering a wide number of clinical metabolomics/exposomics studies (Zhang et al., 2024).

Our targeted metabolomics analysis identified specific metabolites that are significantly dysregulated in the different groups compared in the present work. For example, elevated plasma levels of acylcarnitine C14:2OH and decreased levels of citrulline were significant indicators of survival, whereas elevated plasma levels of CMPF glutaric acid and aspartic acid were associated with positive pneumonia status. These metabolites likely reflect heightened energy demands, immune dysregulation, and organ-specific stress responses induced by SARS-CoV-2.

The identification of altered plasma levels of amino acids, lipids, organic acids, and energy-related metabolites aligns with previous reports indicating that metabolic reprogramming is a core aspect of COVID-19 pathogenesis (Danlos et al., 2021; Lodge et al., 2023; Bruzzone et al., 2023; Martínez-Gómez et al., 2022). Studies have consistently reported disruptions in energy metabolism and immune responses associated with COVID-19 severity, highlighting how metabolites such as acylcarnitines and amino acids signal cellular energy demand and immune modulation. For instance, our observation of elevated plasma acylcarnitine C14:2OH levels in non-survivors mirrors findings by other authors, which linked increased acylcarnitine levels to mitochondrial dysfunction, impaired mitochondrial capacity for fatty acid oxidation and severe outcomes in COVID-19 patients (Mccann et al., 2021). An increase in acylcarnitine levels has also been observed in sepsis (Rogers et al., 2014), where it is associated with increased mortality. Elevation of long-chain acylcarnitines has also been reported in the post-acute phase of moderate infections (Liptak et al., 2022) and in long-COVID patients (Guntur et al., 2022). Decreased levels of citrulline in non-survivors, indicative of immune and endothelial stress, has also been reported by other authors (Tsuge et al., 2024). The increase of lipid-related metabolites in unvaccinated patients may underscore the link between lipid dysregulation and unmitigated viral responses in unvaccinated populations.

Our analysis also emphasizes the role of comorbidities, notably obesity and diabetes, in driving COVID-19-related metabolic changes. The significant dysregulation of triglycerides and glycerophospholipids in patients with these comorbidities aligns with previous studies indicating that metabolic syndrome and obesity exacerbate lipid dysregulation, compromising the immune response and aggravating inflammation. Excessive inflammation can lead to the release of free fatty acids from adipose tissue, either due to tissue damage or rupture, or even by inactivation of lipoprotein lipase (Bagby and Spitzer, 1980). This ultimately results in elevated plasma triglycerides, including triglyceride-rich lipoproteins, which are considered biochemical markers of COVID-19 severity (Rohani-Rasaf et al., 2022). The increase in triglycerides also contributes to greater insulin resistance, raising plasma glucose levels, as observed in this study. In particular, we found that individuals with controlled diabetes and obesity showed higher plasma glucose concentrations than non-diabetic, non-obese individuals (Chang et al., 2022). These metabolic disturbances can further aggravate COVID-19 severity and contribute to a self-perpetuating cycle of chronic diseases misdiagnosis (Yanai, 2020). While multiple mechanisms underline these phenomena, another pathway that can elevate triglycerides in such patients involves inflammation or liver damage. Given the SARS-CoV-2 virus’s affinity for liver tissue, metabolic disruptions in lipid and triglyceride processing may occur, leading to their accumulation in the body (Li et al., 2021; Martinez and Franco, 2021). CMPF was found increased in patients with kidney failure. CMPF is considered a uremic toxin, a harmful metabolite that accumulates in patients with compromised kidney function (Sun et al., 2010; Chen and Chiang, 2021). It is known that lung damage, such as alveolar hemorrhages occurring in pneumonia, is often accompanied by kidney damage, including conditions such as glomerulonephritis (Cárdenas Fernández and Muñoz Palomeque, 2023).

Elderly COVID-19 patients, experiencing heightened inflammatory responses and mitochondrial dysfunction with reduced ATP production, often exhibit increased production of reactive oxygen species (ROS). This increase in ROS depletes glutathione reserves, as glutathione combats the oxidative stress induced by these factors. Consequently, levels of glutamic acid (a component of glutathione) and 5-oxoproline (an intermediate in glutathione synthesis) are also reduced (Páez-Franco et al., 2022). This finding aligns with previous studies showing a decline in these metabolites among elderly COVID-19 patients across various populations (Guarnieri et al., 2023). In general, our analysis reflects that the age and the presence of comorbidities tend to be the most important variables, since more metabolites are implicated in the metabolic dysregulation.

Comparative analyses of different COVID-19 waves showed distinct lipid and amino acid profiles, particularly elevated lipid levels in patients hospitalized during the Delta and Omicron waves, suggesting distinctive metabolic footprints for each SARS-CoV-2 variant. These findings are supported by previous studies that have emphasized the evolving nature of COVID-19’s metabolic impact. The differences in lipid profiles among patients during the Delta and Omicron waves underscore the influence of SARS-CoV-2 variants on host metabolism. Elevated levels of triglycerides, glycerophospholipids, and free fatty acids in Delta and Omicron cases, compared to the Alpha cases, reflect an adaptive metabolic response driven by viral variant pathogenicity and transmission dynamics. Several (non-quantitative) metabolomic studies have been conducted to compare the second COVID-19 wave with subsequent COVID-19 waves. Biagini et al. (2023) found that oxidative stress and inflammation resulting from COVID-19 were highly dependent on the SARS-CoV-2 variant. Their results suggest that the original (wildtype) strain elicited the strongest inflammatory storm, and Omicron significantly differed from previous variants in the levels of pro-inflammatory mediators. The authors found that lipid metabolism was strongly activated by SARS-CoV-2 infection. Both the level and type of oxylipins and polyunsaturated fatty acids (PUFAs) changed significantly across the different COVID-19 waves, suggesting that differences in the virological characteristics of the variants, such as viral load, infectivity, and pathogenicity, as well as immunity from vaccination or prior infection, may also play a role in modulating lipid species changes over time. Additionally, Lewis et al. (2022) compared two waves in the UK (first wave, between May 2020 and July 2020, and second wave between September 2020 and June 2021), and found that while some metabolic changes vary according to each COVID-19 wave, some changes were characteristic of COVID-19 across multiple waves. Recently, Kramaric et al. (2025) by means of untargeted metabolomics found distinctive changes in plasma for infections with Alpha, Delta, and Omicron SARS-CoV2 variants, suggesting that differences could be linked to their relative elicitation of core pathophysiological events associated with COVID-19, for example, inflammation. However, a study with a larger sample size conducted by Ghini et al. (2023) revealed a detailed metabolic analysis of COVID-19 patients using NMR spectroscopy, finding metabolomic and lipoproteomic signatures specific to the disease. The study also investigated sex-specific differences in metabolic responses and across different variants of the virus, although the authors found that the metabolic alterations are not significantly influenced by vaccination status or variant type.

The findings of this study advance our understanding of COVID-19’s clinical and metabolic impacts across different epidemic waves, shedding light on specific metabolic factors that may influence patient outcomes. However, several limitations should be noted. First, the sample size was relatively small (42 patients for Delta and Omicron variants, and 82 for Alpha), which could restrict the generalizability of these findings to broader and more diverse populations. Moreover, we could not systematically determine the variants of each patient through genomic sequencing and the inclusion in each epidemiological wave was done only considering the period of circulation in the country.

Second, this study was conducted exclusively with hospitalized patients in Mexico, potentially limiting the applicability of these results to other regions or ethnic groups. COVID-19 outcomes and metabolomic responses can vary due to clinical, sociodemographic, healthcare access, nutritional status, and genetic backgrounds differences across regions. Furthermore, while the cross-sectional design of this study limits our ability to assess longitudinal changes in metabolomic profiles over time, some dysregulated metabolites, such as amino acids and lipids were clearly identified, resembling behavior in other populations. However, their biological significance remains to be fully elucidated, and further mechanistic studies are required to understand the underlying pathways and clinical implications of these metabolic alterations.

The limited development of quantitative metabolomics techniques applied to clinical settings in Mexico has hindered the characterization of acute COVID-19 versus long COVID-19 patients and the availability of these quantitative variables for the subsequent monitoring of these patients. Only a few studies have reported quantitative metabolomics approaches to characterize COVID-19 infections (López-Hernández et al., 2021; Martínez-Gómez et al., 2022; Herrera-Van Oostdam et al., 2021; Celaya-Padilla et al., 2021; López-Hernández et al., 2023a) while other groups have reported untargeted methods which only determine relative abundances of metabolites (Santana-De Anda et al., 2024; López-Hernández et al., 2023b; Torres-Ruiz et al., 2021; Páez-Franco et al., 2021). This also limits the ability to associate the consequences of the infection with predisposition to diseases such as cancer and autoimmune diseases. As a strength, the present study provides the biggest metabolic characterization of COVID-19 hospitalized patients. The quantitative data of 529 metabolites together with the clinical parameters have been made available for public access (doi:10.17632/sjnv8kjxpb.1). The identification of specific metabolites linked to disease severity and outcomes underlines the potential of metabolomics in enhancing our understanding of COVID-19 pathogenesis. Understanding these metabolic changes can provide valuable insights into personalized therapeutic interventions, as well as long-term monitoring of patients, especially those with risk factors or long COVID. This comprehensive analysis supports the potential use of metabolomics in clinical settings to monitor and predict patient outcomes more accurately. Future research should aim to build on these findings by incorporating larger and more diverse cohorts, as well as longitudinal analyses, to better understand the dynamic metabolic changes over the course of the disease. Such efforts will be instrumental in developing targeted therapeutic strategies and improving patient management in response to COVID-19.

## Data Availability

The datasets presented in this study can be found in online repositories. The names of the repository/repositories and accession number(s) can be found below: https://data.mendeley.com/datasets/sjnv8kjxpb/1 Mendeley repository. DOI: 10.17632/sjnv8kjxpb.1.

## Supplementary material

SUPPLEMENTARY FIGURE S1
Multivariate analysis of plasma metabolome profile of COVID-19 patients from three different waves. **(A)** Score scatter plot based on the PLS-DA comparing patients from Alpha, Delta, and Omicron COVID-19 waves **(B)** Cross validation (10-fold Cross Validation) (Accuracy: 0.93; R^2^: 0.96; Q^2^: 0.89). **(C)** permutation test (1000 permutations).
