# Supplementary material for: Comprehensive clinical and metabolomics profiling of COVID-19 Mexican patients across three epidemiological waves
Source: Front Mol Biosci. 2025 Jun 18;12:1607583. doi: 10.3389/fmolb.2025.1607583 (PMC12214581; doi:10.3389/fmolb.2025.1607583)
Supplement: Supplementary file 1 [file Table7.docx]

**Table S7.** Dysregulated metabolites in obesity and diabetes clustering.

| **Metabolite** | **Classes** | **p-value** |
| --- | --- | --- |
| TG(16:1_38:4) | Triglycerides | **0.0005** |
| TG(16:1_38:3) | Triglycerides | **0.0006** |
| TG(17:0_36:3) | Triglycerides | **0.0031** |
| TG(18:1_35:2) | Triglycerides | **0.0039** |
| TG(16:0_35:3) | Triglycerides | **0.0042** |
| TG(18:1_38:7) | Triglycerides | **0.0049** |
| TG(18:1_33:2) | Triglycerides | **0.0050** |
| TG(16:0_40:6) | Triglycerides | **0.0051** |
| TG(16:1_38:5) | Triglycerides | **0.0054** |
| TG(18:1_34:3) | Triglycerides | **0.0057** |
| TG(17:1_36:3) | Triglycerides | **0.0068** |
| TG(20:5_36:3) | Triglycerides | **0.0076** |
| LysoPC a C28:0 | Glycerophospholipids | **0.0081** |
| TG(18:1_35:3) | Triglycerides | **0.0084** |
| TG(18:0_34:3) | Triglycerides | **0.0089** |
| TG(16:0_37:3) | Triglycerides | **0.0092** |
| TG(18:2_33:0) | Triglycerides | **0.0093** |
| TG(18:2_32:2) | Triglycerides | **0.0098** |
| TG(18:0_36:1) | Triglycerides | **0.0099** |
| TG(18:2_35:1) | Triglycerides | 0.0101 |
| TG(18:1_32:3) | Triglycerides | 0.0105 |
| Propionic acid | Organic acids | 0.0105 |
| TG(14:0_36:3) | Triglycerides | 0.0110 |
| TG(14:0_36:2) | Triglycerides | 0.0111 |
| TG(20:2_34:1) | Triglycerides | 0.0116 |
| TG(22:4_34:2) | Triglycerides | 0.0116 |
| TG(17:0_34:3) | Triglycerides | 0.0116 |
| TG(14:0_38:4) | Triglycerides | 0.0123 |
| TG(16:0_38:4) | Triglycerides | 0.0123 |
| Malic acid | Organic acids | 0.0128 |
| TG(18:1_34:4) | Triglycerides | 0.0135 |
| TG(16:0_38:2) | Triglycerides | 0.0136 |
| TG(18:0_32:2) | Triglycerides | 0.0136 |
| TG(17:1_34:2) | Triglycerides | 0.0136 |
| TG(18:1_36:1) | Triglycerides | 0.0137 |
| TG(18:1_34:2) | Triglycerides | 0.0137 |
| TG(20:3_36:3) | Triglycerides | 0.0138 |
| TG(22:5_32:0) | Triglycerides | 0.0139 |
| TG(18:1_38:5) | Triglycerides | 0.0148 |
| TG(20:4_32:1) | Triglycerides | 0.0149 |
| TG(18:2_30:1) | Triglycerides | 0.0158 |
| TG(16:1_36:2) | Triglycerides | 0.0165 |
| TG(16:1_36:5) | Triglycerides | 0.0166 |
| TG(20:2_34:3) | Triglycerides | 0.0167 |
| TG(16:1_36:3) | Triglycerides | 0.0185 |
| TG(18:1_34:1) | Triglycerides | 0.0187 |
| TG(16:0_40:7) | Triglycerides | 0.0187 |
| TG(16:0_34:3) | Triglycerides | 0.0189 |
| TG(18:3_32:0) | Triglycerides | 0.0192 |
| TG(16:0_36:2) | Triglycerides | 0.0192 |
| TG(20:1_34:0) | Triglycerides | 0.0195 |
| TG(18:1_32:2) | Triglycerides | 0.0204 |
| TG(18:2_33:1) | Triglycerides | 0.0206 |
| TG(16:0_38:3) | Triglycerides | 0.0207 |
| TG(16:0_35:2) | Triglycerides | 0.0209 |
| TG(16:0_34:2) | Triglycerides | 0.0220 |
| PC aa C26:0 | Glycerophospholipids | 0.0220 |
| TG(20:1_34:3) | Triglycerides | 0.0229 |
| TG(16:0_30:2) | Triglycerides | 0.0231 |
| TG(18:0_34:2) | Triglycerides | 0.0235 |
| TG(20:5_34:1) | Triglycerides | 0.0242 |
| TG(16:0_38:6) | Triglycerides | 0.0248 |
| TG(20:3_36:4) | Triglycerides | 0.0249 |
| TG(16:0_38:5) | Triglycerides | 0.0254 |
| TG(18:1_36:0) | Triglycerides | 0.0256 |
| Fumaric acid | Organic acids | 0.0262 |
| TG(16:0_38:7) | Triglycerides | 0.0267 |
| TG(20:1_34:2) | Triglycerides | 0.0279 |
| TG(16:1_36:4) | Triglycerides | 0.0281 |
| alpha-Ketoglutaric acid | Organic acids | 0.0281 |
| TG(16:0_28:2) | Triglycerides | 0.0284 |
| TG(14:0_36:1) | Triglycerides | 0.0284 |
| TG(20:2_34:2) | Triglycerides | 0.0288 |
| TG(16:1_36:1) | Triglycerides | 0.0299 |
| TG(18:2_34:0) | Triglycerides | 0.0301 |
| TG(20:1_34:1) | Triglycerides | 0.0304 |
| TG(18:2_34:4) | Triglycerides | 0.0305 |
| TG(16:0_38:1) | Triglycerides | 0.0305 |
| TG(18:2_38:5) | Triglycerides | 0.0314 |
| TG(14:0_36:4) | Triglycerides | 0.0316 |
| TG(20:3_34:1) | Triglycerides | 0.0328 |
| TG(14:0_34:0) | Triglycerides | 0.0337 |
| TG(18:2_34:1) | Triglycerides | 0.0345 |
| TG(18:2_32:1) | Triglycerides | 0.0350 |
| C18:1 | Acylcarnitines | 0.0354 |
| TG(18:3_34:0) | Triglycerides | 0.0359 |
| TG(20:3_34:2) | Triglycerides | 0.0363 |
| TG(18:1_33:0) | Triglycerides | 0.0365 |
| TG(20:3_34:3) | Triglycerides | 0.0366 |
| TG(20:5_34:2) | Triglycerides | 0.0366 |
| TG(22:5_34:2) | Triglycerides | 0.0372 |
| TG(20:2_32:0) | Triglycerides | 0.0373 |
| TG(18:3_32:1) | Triglycerides | 0.0380 |
| Pyruvic acid | Organic acids | 0.0381 |
| Hex3Cer(d18:1/16:0) | Glycosphingolipids | 0.0391 |
| TG(18:2_32:0) | Triglycerides | 0.0394 |
| TG(18:2_28:0) | Triglycerides | 0.0396 |
| TG(16:0_36:3) | Triglycerides | 0.0399 |
| TG(20:1_32:2) | Triglycerides | 0.0399 |
| TG(18:1_28:1) | Triglycerides | 0.0404 |
| TG(16:1_34:1) | Triglycerides | 0.0407 |
| TG(18:1_36:2) | Triglycerides | 0.0414 |
| TG(16:0_34:4) | Triglycerides | 0.0414 |
| TG(18:0_38:7) | Triglycerides | 0.0415 |
| TG(18:2_38:4) | Triglycerides | 0.0416 |
| TG(16:1_34:2) | Triglycerides | 0.0419 |
| TG(14:0_34:3) | Triglycerides | 0.0421 |
| DG(16:1_18:2) | Diglycerides | 0.0424 |
| DG(17:0_18:1) | Diglycerides | 0.0432 |
| TG(18:1_33:1) | Triglycerides | 0.0437 |
| TG(18:1_30:2) | Triglycerides | 0.0438 |
| TG(20:1_32:1) | Triglycerides | 0.0439 |
| TG(16:0_34:1) | Triglycerides | 0.0442 |
| TG(17:1_34:3) | Triglycerides | 0.0448 |
| TG(18:1_30:1) | Triglycerides | 0.0467 |
| TG(18:2_35:2) | Triglycerides | 0.0477 |
| TG(14:0_34:2) | Triglycerides | 0.0482 |
| TG(18:0_36:2) | Triglycerides | 0.0492 |
| TG(18:1_32:1) | Triglycerides | 0.0494 |
| TG(20:0_34:1) | Triglycerides | 0.0495 |

Significant values (p ≤ 0.01) are highlighted in bold.
