# Supplementary material for: Comprehensive clinical and metabolomics profiling of COVID-19 Mexican patients across three epidemiological waves
Source: Front Mol Biosci. 2025 Jun 18;12:1607583. doi: 10.3389/fmolb.2025.1607583 (PMC12214581; doi:10.3389/fmolb.2025.1607583)
Supplement: Supplementary file 2 [file Table2.docx]

**Table S2.** Dysregulated metabolites in the vaccination clustering.

| **Metabolite** | **Classes** | **p-value** |
| --- | --- | --- |
| TG(14:0_32:2) | Triglycerides | **0.0100** |
| Hex2Cer(d18:1/18:0) | Glycosylceramides | 0.0112 |
| TG(18:1_28:1) | Triglycerides | 0.0172 |
| TG(18:2_31:0) | Triglycerides | 0.0196 |
| TG(18:0_30:0) | Triglycerides | 0.0200 |
| TG(14:0_35:1) | Triglycerides | 0.0213 |
| TG(17:0_32:1) | Triglycerides | 0.0213 |
| Malic acid | Organic acids | 0.0281 |
| TG(16:0_28:2) | Triglycerides | 0.0330 |
| Fumaric acid | Organic acids | 0.0424 |

Significant values (p ≤ 0.01) are highlighted in bold.
