# Supplementary material for: Comprehensive clinical and metabolomics profiling of COVID-19 Mexican patients across three epidemiological waves
Source: Front Mol Biosci. 2025 Jun 18;12:1607583. doi: 10.3389/fmolb.2025.1607583 (PMC12214581; doi:10.3389/fmolb.2025.1607583)
Supplement: Supplementary file 3 [file Table5.docx]

**Table S5.** Dysregulated metabolites in gender clustering.

| **Metabolite** | **Classes** | **p-value** |
| --- | --- | --- |
| alpha-Aminoadipic acid | L-alpha-amino acids | **0.0094** |
| Cer(d18:1/16:0) | Ceramides | **0.0100** |
| Glyceric acid | Organic acids | 0.0196 |
| TG(20:0_34:1) | Triglycerides | 0.0232 |
| C3 | Acylcarnitines | 0.0264 |
| TG(20:1_32:2) | Triglycerides | 0.0268 |
| TG(18:0_36:1) | Triglycerides | 0.0299 |
| Phenylacetic acid | Organic acids | 0.0300 |
| TG(18:1_36:0) | Triglycerides | 0.0325 |
| Propionic acid | Organic acids | 0.0348 |
| HexCer(d18:1/22:0) | Glycosylceramides | 0.0384 |
| Aspartic acid | Amino Acids | 0.0390 |
| TG(18:2_36:0) | Triglycerides | 0.0440 |
| Methylmalonic acid | Organic acids | 0.0458 |
| Cer(d18:1/18:0) | Ceramides | 0.0476 |
| HexCer(d18:1/18:0) | Glycosylceramides | 0.0491 |
| N-Acetyl-Glycine | N-acyl-alpha amino acids | 0.0497 |

Significant values (p ≤ 0.01) are highlighted in bold.
