# Supplementary material for: Comprehensive clinical and metabolomics profiling of COVID-19 Mexican patients across three epidemiological waves
Source: Front Mol Biosci. 2025 Jun 18;12:1607583. doi: 10.3389/fmolb.2025.1607583 (PMC12214581; doi:10.3389/fmolb.2025.1607583)
Supplement: Supplementary file 4 [file Table3.docx]

**Table S3.** Dysregulated metabolites in survival clustering.

| **Metabolite** | **Classes** | **p-value** |
| --- | --- | --- |
| C14:2OH | Acylcarnitines | **0.0056** |
| Citrulline | L-alpha-amino acids | **0.0056** |
| Cer(d18:1/18:0) | Ceramides | 0.0143 |
| PC ae C38:5 | Glycerophospholipids | 0.0154 |
| PC ae C44:5 | Glycerophospholipids | 0.0160 |
| PC ae C44:6 | Glycerophospholipids | 0.0162 |
| PC ae C36:4 | Glycerophospholipids | 0.0168 |
| Glucose | Sugars | 0.0184 |
| C14 | Acylcarnitines | 0.0204 |
| HexCer(d18:2/23:0) | Glycosylceramides | 0.0242 |
| TG(18:1_36:0) | Triglycerides | 0.0295 |
| Cer(d18:1/22:0) | Ceramides | 0.0296 |
| PC aa C36:0 | Glycerophospholipids | 0.0311 |
| PC ae C36:5 | Glycerophospholipids | 0.0333 |
| C8 | Acylcarnitines | 0.0401 |
| PC aa C36:1 | Glycerophospholipids | 0.0412 |
| SM C24:1 | Sphingomyelins | 0.0423 |
| Aspartic acid | Amino Acids | 0.0468 |
| PC aa C42:0 | Glycerophospholipids | 0.0497 |

Significant values (p ≤ 0.01) are highlighted in bold.
