# Supplementary material for: Comprehensive clinical and metabolomics profiling of COVID-19 Mexican patients across three epidemiological waves
Source: Front Mol Biosci. 2025 Jun 18;12:1607583. doi: 10.3389/fmolb.2025.1607583 (PMC12214581; doi:10.3389/fmolb.2025.1607583)
Supplement: Supplementary file 6 [file Table4.docx]

**Table S4.** Dysregulated metabolites in pneumonia clustering.

| **Metabolite** | **Classes** | **p-value** |
| --- | --- | --- |
| CMPF | Fatty acids | **0.0054** |
| Glutaric acid | Organic acids | **0.0060** |
| Aspartic acid | Amino acids | **0.0100** |
| HexCer(d18:1/18:1) | Glycosylceramides | 0.0103 |
| Malic acid | Organic acids | 0.0130 |
| Butyric acid + Isobutyric acid | Fatty Acids | 0.0160 |
| 2-hydroxyglutaric acid | Short-chain hydroxy acids | 0.0210 |
| 2-Hydroxyisobutyric acid | Organic acids | 0.0231 |
| C12:1 | Acylcarnitines | 0.0260 |
| TG(16:0_34:0) | Triglycerides | 0.0293 |
| N-Acetyl-Glycine | N-acyl-alpha amino acids | 0.0300 |
| HexCer(d18:2/16:0) | Glycosylceramides | 0.0306 |
| Glycine | Amino Acids | 0.0307 |
| TG(20:4_36:3) | Triglycerides | 0.0319 |
| TG(18:1_32:0) | Triglycerides | 0.0320 |
| TG(20:4_34:3) | Triglycerides | 0.0322 |
| C14:2 | Acylcarnitines | 0.0336 |
| C14:1OH | Acylcarnitines | 0.0341 |
| TG(20:1_34:0) | Triglycerides | 0.0361 |
| Fumaric acid | Organic acids | 0.0363 |
| Propionic acid | Organic acids | 0.0364 |
| Orotic acid | Organic acids | 0.0398 |
| TG(14:0_34:1) | Triglycerides | 0.0428 |
| TG(16:0_34:1) | Triglycerides | 0.0432 |
| Methionine sulfoxide | L-alpha-amino acids | 0.0441 |
| TG(18:1_30:0) | Triglycerides | 0.0461 |
| TG(18:0_32:0) | Triglycerides | 0.0477 |
| TG(20:1_32:1) | Triglycerides | 0.0488 |

Significant values (p ≤ 0.01) are highlighted in bold.
