# Supplementary material for: Comprehensive clinical and metabolomics profiling of COVID-19 Mexican patients across three epidemiological waves
Source: Front Mol Biosci. 2025 Jun 18;12:1607583. doi: 10.3389/fmolb.2025.1607583 (PMC12214581; doi:10.3389/fmolb.2025.1607583)
Supplement: Supplementary file 7 [file Table6.docx]

**Table S6.** Dysregulated metabolites in age clustering.

| **Metabolite** | **Classes** | **p-value** |
| --- | --- | --- |
| Hypoxanthine | Nucleobases | **0.0015** |
| 5-Oxoproline | Alpha amino acids | **0.0054** |
| Glutamic acid | Amino Acids | **0.0054** |
| PC aa C42:2 | Glycerophospholipids | **0.0056** |
| PC ae C42:1 | Glycerophospholipids | **0.0069** |
| Pipecolic acid | Alpha amino acids | **0.0069** |
| PC aa C40:3 | Glycerophospholipids | **0.0071** |
| PC aa C40:4 | Glycerophospholipids | 0.0112 |
| DG(16:0_16:1) | Diglycerides | 0.0171 |
| PC aa C42:5 | Glycerophospholipids | 0.0195 |
| 3-Hydroxyisobutyric acid | Organic acids | 0.0207 |
| C18:1 | Acylcarnitines | 0.0225 |
| PC aa C36:6 | Glycerophospholipids | 0.0246 |
| Aspartic acid | Amino Acids | 0.0257 |
| PC ae C40:3 | Glycerophospholipids | 0.0264 |
| PC ae C42:2 | Glycerophospholipids | 0.0267 |
| PC ae C44:3 | Glycerophospholipids | 0.0295 |
| PC aa C40:5 | Glycerophospholipids | 0.0310 |
| C7DC | Acylcarnitines | 0.0350 |
| PC ae C38:1 | Glycerophospholipids | 0.0389 |
| PC aa C38:1 | Glycerophospholipids | 0.0400 |
| Glutamine | Amino Acids | 0.0406 |
| PC aa C40:2 | Glycerophospholipids | 0.0410 |
| PC aa C38:4 | Glycerophospholipids | 0.0417 |
| Cystathionine | l-cysteine-s-conjugates | 0.0418 |
| PC ae C40:1 | Glycerophospholipids | 0.0437 |
| PC ae C38:0 | Glycerophospholipids | 0.0440 |
| TG(16:0_35:2) | Triglycerides | 0.0471 |
| PC ae C38:3 | Glycerophospholipids | 0.0481 |
| TG(18:1_36:0) | Triglycerides | 0.0486 |

Significant values (p ≤ 0.01) are highlighted in bold.
