# Supplementary figures and images for: Comprehensive clinical and metabolomics profiling of COVID-19 Mexican patients across three epidemiological waves
Source: Front Mol Biosci. 2025 Jun 18;12:1607583. doi: 10.3389/fmolb.2025.1607583 (PMC12214581; doi:10.3389/fmolb.2025.1607583)

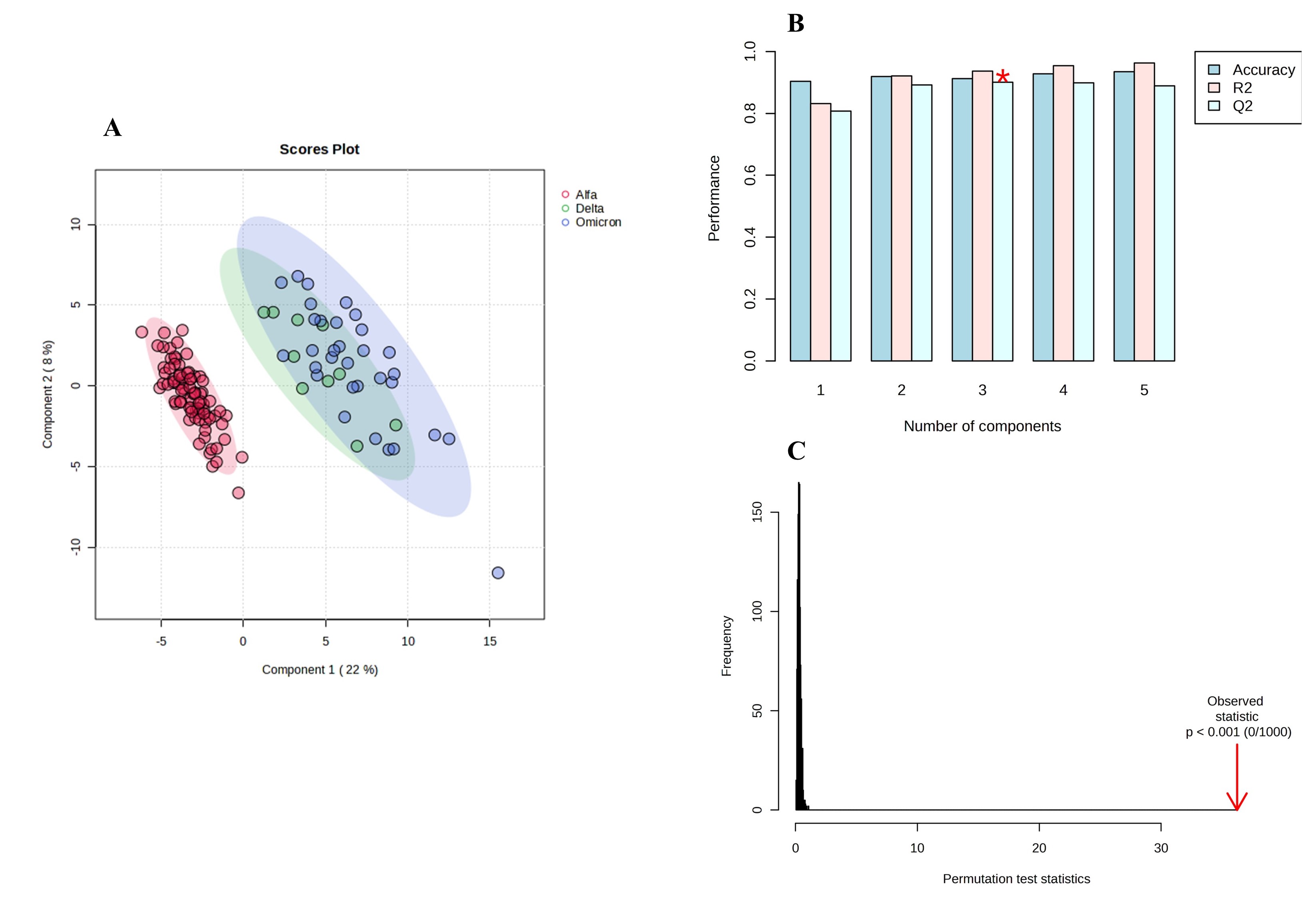

Supplement: Supplementary file 8 [file Image1.jpg]
